# The Mapping Between Transformed Reaction Time Costs and Models of Processing in Aging and Cognition

**DOI:** 10.1037/pag0000298

**Published:** 2018-10-08

**Authors:** Craig Hedge, Georgina Powell, Petroc Sumner

**Affiliations:** 1 School of Psychology, Cardiff University

**Keywords:** aging, reaction times, diffusion model, slowing, proportional RT costs

## Abstract

Older adults tend to have slower response times (RTs) than younger adults on cognitive tasks. This makes the examination of domain-specific deficits in aging difficult, as differences between conditions in raw RTs (RT costs) typically increase with slower average RTs. Here, we examine the mapping between 2 established approaches to dealing with this confound in the literature. The first is to use transformed RT costs, with the *z*-score and proportional transforms both being commonly used. The second is to use mathematical models of choice RT behavior, such as the drift-diffusion model (Ratcliff, 1978). We simulated data for younger and older adults from the drift-diffusion model under 4 scenarios: (a) a domain specific deficit, (b) general slowing, (c) strategic slowing, and (d) a slowing of nondecision processes. In each scenario we varied the size of the difference between younger and older adults in the model parameters, and examined corresponding effect sizes and Type I error rates in the raw and transformed RT costs. The *z*-score transformation provided better control of Type I error rates than the raw or proportional costs, though did not fully control for differences in the general slowing and strategic slowing scenarios. We recommend that RT analyses are ideally supplemented by analyses of error rates where possible, as these may help to identify the presence of confounds. To facilitate this, it would be beneficial to include conditions that elicit below ceiling accuracy in tasks.

It has been well established that elderly people are typically slower on choice RT tasks compared with younger adults (Anstey, Dear, Christensen, & Jorm, 2005; Bugg, Zook, DeLosh, Davalos, & Davis, 2006; Salthouse, 1985, 1996). Further, there is a great deal of research examining whether older adults show deficits in specific domains, such as response inhibition or executive functioning, compared with younger adults (e.g., Castel, Balota, Hutchison, Logan, & Yap, 2007; van der Lubbe & Verleger, 2002). However, there are discrepancies in the literature as to whether observed effects reflect domain specific deficits, or if differences can be accounted for by general processing speed (Verhaeghen, 2011).

In widely used tasks such as the Stroop task (Stroop, 1935) or task switching, RTs in a baseline condition are subtracted from a condition that requires additional processing, producing an RT cost. In within-subject studies, the magnitude of the RT cost is interpreted as an index of the process of interest, such as the time taken to resolve conflict or switch task sets. However, such an interpretation is confounded when comparing groups that differ in their overall response speed, as RT costs generally increase with slower RTs (Faust, Balota, Spieler, & Ferraro, 1999).The potential contamination of task specific effects has led to different methods being used to control for general slowing in aging, the appropriateness of which have been the subject of much discussion in the literature (e.g., Cerella, 1991; Faust et al., 1999; Myerson, Adams, Hale, & Jenkins, 2003; Ratcliff, Spieler, & McKoon, 2000; Salthouse & Hedden, 2002).

Here, we examine the mapping between two different kinds of approach—RT transformations and decision models. We focus on two commonly used RT transformations. The first is to take proportional RT costs, in which the raw RT cost is divided by the mean RT in the baseline condition (e.g., Bialystok, Craik, & Luk, 2008; Colcombe, Kramer, Erickson, & Scalf, 2005; de Bruin & Della Sala, 2018; Gold, Kim, Johnson, Kryscio, & Smith, 2013; Gratton, Wee, Rykhlevskaia, Leaver, & Fabiani, 2009; Henry et al., 2015; Kousaie & Phillips, 2012; Lawo & Koch, 2014; Mazaheri, Roerdink, Duysens, Beek, & Peper, 2016; Truong & Yang, 2014; Weissberger, Wierenga, Bondi, & Gollan, 2012; Yang & Hasher, 2007; Zhu, Zacks, & Slade, 2010). The second transformation is the *z*-score, in which the overall mean RT is subtracted from each trial RT, and the result divided by the overall *SD*. The resultant z-transformed values can then be averaged per condition, and a cost calculated (e.g., Aschenbrenner & Balota, 2017; Bush, Hess, & Wolford, 1993; Christ, White, Mandernach, & Keys, 2001; Faust et al., 1999; Hummert, Garstka, O’Brien, Greenwald, & Mellott, 2002; Madden et al., 2014; Paxton, Barch, Racine, & Braver, 2008). Analyses of the transformed costs are typically performed in addition to, or in place of, the analysis of raw RT costs, to control for group differences in overall RTs. The use of both methods is not specific to any paradigm or cognitive domain, nor is it restricted to studies of aging (e.g., Pe, Koval, & Kuppens, 2013; Schmitter-Edgecombe & Langill, 2006; Sumner, Edden, Bompas, & Singh, 2010; von Bastian, Souza, & Gade, 2016). The *z*-score and proportional transformations assume different quantitative relationships between overall RT and the magnitude of the RT costs, however, these assumptions are rarely explicitly justified (though see Faust et al., 1999).

Evaluating the assumptions of an RT transformation is not a trivial task, as it requires knowledge of the way in which RTs map on to the cognitive processes that generate them. For this purpose, mathematical models of choice RT behavior provide a potentially valuable reference, as they explicitly specify the relationship between behavior and the theorized underlying mechanisms. We used the drift diffusion model (DDM; Ratcliff, 1978; Ratcliff & Rouder, 1998) to simulate four hypothetical scenarios that could affect RT costs and/or average RTs, based on parameter values that have been reported in a study of younger and older adults. These scenarios correspond to a domain specific deficit, general slowing, strategic slowing, and a slowing of perceptual-motor (i.e., nondecision) processes. If the RT transformations can be mapped specifically to domain specific deficits in the DDM, we would expect them to show group differences only in the domain specific scenario. In other words, we can consider a group difference observed in the transformed costs in scenarios of general slowing, strategic slowing and perceptual-motor slowing to be a Type I error (false positive). To anticipate the results of our simulations, the *z*-score transformation showed a lower Type I error rate than the raw and proportional costs, though it still exceeded the nominal level (5%) in the presence of general slowing and strategic slowing. Counterintuitively, the proportional costs can even *create* an apparent advantage for older adults in the presence of slower RTs that actually arise from perceptual or motor slowing.

## The Drift Diffusion Model

The DDM is one of a group of models developed to account for both the speed and accuracy of performance on choice RT tasks (see also Brown & Heathcote, 2008; Carpenter & Williams, 1995; Usher & McClelland, 2001). These models differ slightly in the assumptions and construction, but for our current purposes they all produce similar behavior (cf. Donkin, Brown, Heathcote, & Wagenmakers, 2011). For comparison, we conduct a simulation using an alternative model, the Linear Ballistic Accumulator (Brown & Heathcote, 2008), in supplementary material B.

In a two-choice RT task, the DDM assumes that on each trial a decision mechanism samples evidence for one or the other option over time. This continues until a criterion level of evidence is reached for one of the options, at which point the motor response is initiated. Researchers are typically interested in three key parameters. First, drift rate (v) is the average rate at which evidence is accumulated. This typically varies between conditions, such that trials in a relatively easy condition would have a higher mean drift rate compared with a harder condition. The lower drift rate in harder trials accounts for their slower RTs and typically lower accuracy rates. The second parameter of interest is boundary separation (a), which refers to the level of evidence that an individual requires for a response. Individuals who are very cautious will set a high threshold, so they make fewer errors at the expense of having longer RTs. Where trials are randomly intermixed within blocks, it is typically assumed that boundary separation does not differ between conditions. Finally, nondecision time (Ter) is included to account for the speed of visual processing and motor implementation. As with boundary separation, it is typically assumed that nondecision time does not vary between conditions when they are randomly intermixed.

The drift-diffusion model has now been applied to the study of aging across a wide range of cognitive domains (McKoon & Ratcliff, 2013; Ratcliff & McKoon, 2015; Ratcliff, Thapar, Gomez, & McKoon, 2004; Ratcliff, Thapar, & McKoon, 2006a, 2006b, 2011; Schuch, 2016; Spaniol, Madden, & Voss, 2006; Starns & Ratcliff, 2010; Thapar, Ratcliff, & McKoon, 2003). A consistent finding from this literature is that older adults often show increased boundary separation, and prolonged nondecision time. The evidence for differences in drift rates between younger and older adults is mixed, and varies between tasks (Ratcliff et al., 2006a; Verhaeghen, 2014), which has been used as an argument against a global deficit in information processing in older adults.

## Four Scenarios Leading to Changes in RTs

**Figure 1 fig1:**
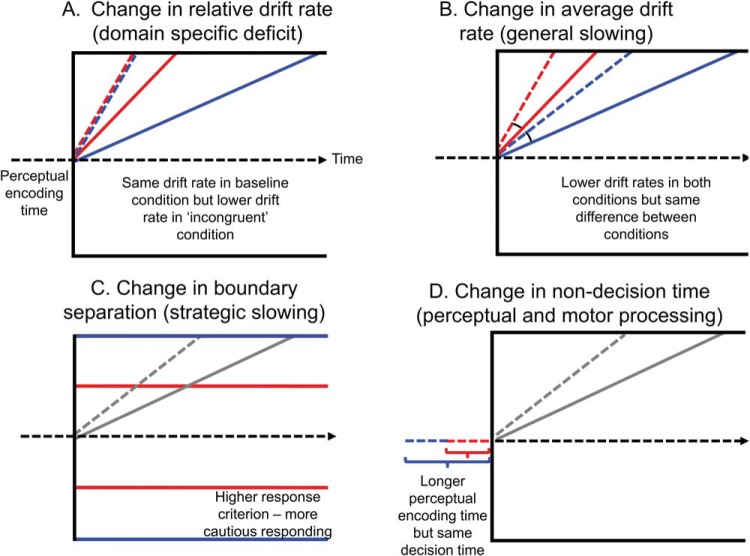
Four scenarios in which two individuals could produce different response times (RTs) in the drift diffusion model. In all cases, the individual that would produce slower RTs is portrayed in blue (Dark grey), and the faster individual is shown in red (Light grey). (A) Represents the scenario in which most researchers are typically interested. In this scenario, both individuals produce the same drift rates in the baseline condition (red and blue dashed lines), but one shows a domain specific deficit, in which the drift rate in the more difficult condition is lower. (B) Global slowing, reflecting lower mean drift rates in both conditions while maintaining the same difference between drift rates in both fast and slow individuals (note that the angular difference is unchanged). (C) A change in boundary separation or strategic slowing. The individual represented by the red line requires less evidence to make a response, resulting in faster RTs (and more errors). (D) A change in nondecision time, reflecting a longer period of perceptual encoding in the individual with slower RTs. The decision phase (boundary separation and drift rates are unchanged.

Using the framework of the DDM, we can create differences between two hypothetical individuals (or groups of individuals) in mean RTs and mean RT costs by varying parameters of the model that correspond to different sources of slowing. These scenarios are illustrated in Figure 1. In each case, the individual who produces slower RTs in one or both conditions is shown in blue, and the faster individual in red.[Fig-anchor fig1]

The first scenario (Figure 1A) depicts two individuals whose drift rate in the baseline condition is equivalent, but who differ in their drift rates in the more difficult condition. This is how a domain specific deficit would be implemented in the DDM—the individual portrayed in blue is less able to process the stimulus in the presence of increased difficulty, distraction, or interference. The second scenario portrays a global change in information processing speed in the absence of a domain specific effect. This can be characterized in the context of the DDM by a decrease in the drift rates for both conditions *while maintaining the same difference between conditions* (see Figure 1B). In the third scenario (Figure 1C), the blue individual has a greater boundary separation compared with the red individual, meaning they wait for more evidence before responding in both conditions (i.e., they are more cautious). In the final scenario (Figure 1D), the individual in blue is slower because of a prolonged period of perceptual encoding before the decision process (a prolonged motor output time would have the same effect).

Note that studies of the effect of aging in particular cognitive domains are typically interested in the differences reflected in Scenario A. Scenario B most closely reflects what the proportional and *z*-score transforms are used to control for.

## Simulated Behavioral Costs

Given the four scenarios outlined in Figure 1, we can simulate data for younger and older adults in two conditions of differing difficulty to assess the way in which changes in these underlying parameters affect raw RT costs, transformed RT costs, and error costs. We did this for a range of effect sizes for the difference between younger and older adults, to assess whether the size of confounding effects influenced the effectiveness of the transforms. In each scenario, we simulated pools of 2,000 younger and older adults with 10,000 trials per condition, so as to remove the influence of noise in our estimates. To obtain plausible ranges for a choice RT task in our simulations, we derived parameters from fits of the DDM to a lexical-decision task in younger and older adults by Ratcliff, Thapar, et al., 2004, their Tables 3 and 4) and a previous simulation article (van Ravenzwaaij & Oberauer, 2009). These values are representative of those reported across a range of tasks (Ratcliff, Thapar, Smith, & McKoon, 2005, 2006a). Our “easy” and “hard” condition drift rates were informed by fitted drift rates for high and low frequency words.

The values used are shown in Table 1. Group differences were simulated in each scenario by changing the mean of the relevant parameter for older adults to reflect five different standardized effect sizes (Cohen’s *d* of .2 to 1.4 in intervals of .3, where *d* = mean difference/pooled *SD*).[Table-anchor tbl1]

Note that Cohen’s *d*s of .2, .5, and .8 are traditionally considered to be small, medium, and large effect sizes, respectively (Cohen, 1992). We discuss the plausibility of parameter differences of these magnitudes in the discussion. In each scenario, we simulated parameter values from a normal distribution with a common *SD*. Drift rates for easy and hard trials were generated from a multivariate normal distribution (using Matlab’s mvnrnd function), which generates two normally distributed random variables with specified means, variances, and covariance. Following van Ravenzwaaij and Oberauer (2009), we assumed a correlation of .8 between easy and hard drift rates, reflecting the observation that performance across conditions is typically highly correlated. As a mean drift rate of 0 would produce chance accuracy, we truncated values to a minimum of .1 (at most, this meant replacing 5.4% of older adult’s hard drift rates in the largest general slowing effect size scenario). For simplicity, we assumed a common *SD* (.07) for both drift rates, as in van Ravenzwaaij and Oberauer. This is slightly smaller than the pooled *SD* from Ratcliff, Thapar, et al.’s (2004) fits, so as to minimize the number of values that needed to be truncated at the larger effect sizes.

We used a common value for between-trial variability in drift rates (η = .1) for both groups in all simulations. The mean starting point of the diffusion process was fixed to a/2 for all simulations. Starting point variability and nondecision time variability were fixed at zero. Data were simulated using the DMAT toolbox (Vandekerckhove & Tuerlinckx, 2008) in Matlab (2014; The MathWorks Inc., Natick, MA).

The calculation of mean RTs excluded incorrect responses. The proportional RT cost was calculated as (hard RT-easy RT)/easy RT. *z*-score RT costs were calculated by subtracting the mean RT of all trials from each individual RT, and dividing by the *SD* of RTs across all trials. The transformed values were then averaged in each condition, and a cost calculated from the resultant condition means. Descriptive statistics for the smallest and largest effect size are shown in Table 2, while a detailed summary is reported in supplementary material A. These ranges are similar to those used in previous discussions of RT transformations (Faust et al., 1999; Hale, Myerson, Faust, & Fristoe, 1995).[Table-anchor tbl2]

**Figure 2 fig2:**
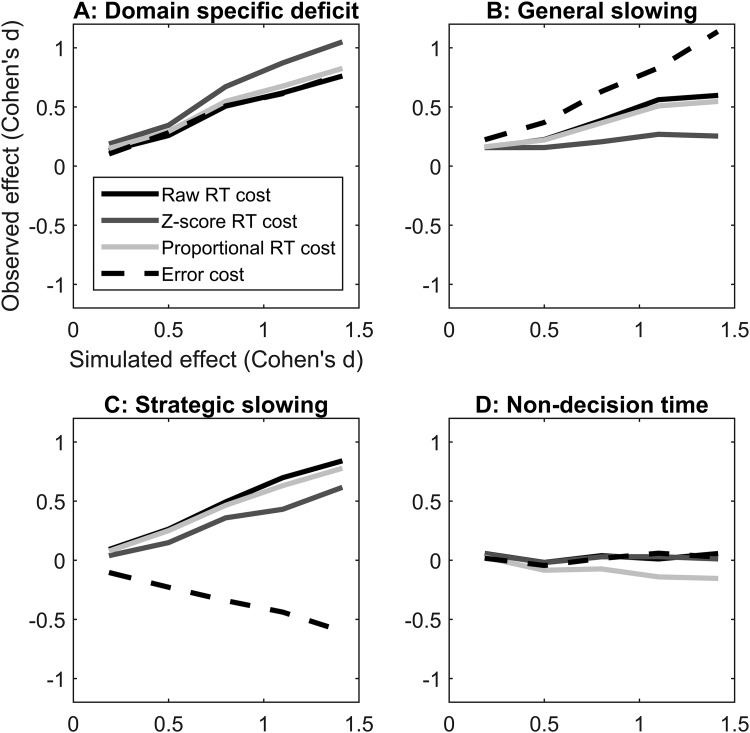
Relationship between the effect size in diffusion model parameters manipulated in each scenario (*x*-axis) and the effect size observed in the behavioral measures derived from the simulated data (*y*-axis). Positive effect sizes on the *y*-axis indicate larger costs in the older adult group. See Table 1 and Figure 1 for parameters manipulated in each scenario. The effect sizes are nonzero for all raw and transformed costs in Scenarios B and C, and for proportional response time (RT) costs in Scenario D. This indicates that they do not control for group differences in these confounding parameters.

To assess the way in which studies with plausible sample sizes would be affected by group differences in each scenario and effect size, we randomly sampled from the pools of simulated participants to create 5,000 pseudoexperiments with *N* = 30 per group. For each pseudo experiment we calculated the mean RT, RT cost, proportional RT cost, *z*-score cost and error cost and tested whether the group difference was significant (*p* < .05) in an independent *t* test. Figure 2 plots the average effect size (younger vs. older adults) for the behavioral costs in relation to the effect size of the difference in the underlying parameters in each scenario. If the *z*-score and proportional RT costs control for the confounding factors, the lines should be flat in Scenarios B to D. We also report the percentage of pseudoexperiments in which the group difference was significant according to this traditional criterion in Table 3. As only Scenario A simulates an underlying domain-specific deficit, the percentages for Scenarios B to D can be interpreted as Type I error rates.[Fig-anchor fig2][Table-anchor tbl3]

In Scenario A, reflecting a domain specific deficit, the effect size in each of the behavioral costs increases with the underlying manipulation exactly as it should do because all the measures are expected to capture domain specific deficits. Scenario B reflects global slowing in older adults. Here, the simulated older adults are less efficient in processing evidence in both conditions compared with the simulated younger adults, but have the same relative difference between conditions. Ideally transformed data should minimize effects here, so they are not confused with domain specific effects (Scenario A). All the behavioral costs show some sensitivity to this general slowing, with increased false positive rates associated with larger effect sizes. However, while the proportional RT cost shows little improvement over raw RT costs, the *z*-score transformation does decrease the false positive rate in this scenario.

Scenario C reflects differences in boundary separation (response caution/strategic slowing). The older adult groups have a higher boundary separation, such that they wait for more evidence before making a response. In this scenario, younger and older adults have identical drift rates for easy and hard trials, however, older adults have larger RT costs because the RT difference scales with higher levels of response caution (cf. Ratcliff et al., 2000). Critically, the transformed costs do not correct for this, and the “deficit” is apparent here too. Further, the strategic slowing in older adults leads to relatively smaller error costs. Thus, one would draw different conclusions about the relative ability of younger and older adults if we were to use RT costs or error costs in this scenario (see Hedge et al., in press for an extended discussion of this point). Finally, in Scenario D, the simulated older adults have a longer nondecision time compared with younger adults. In the simplest form, nondecision time is a constant that is added to the RTs for both conditions, so this did not affect the variance of RTs or the difference between conditions in our simulations. This means that the absolute RT costs, and error cost are identical in both groups. The *z*-score is also insensitive to this change, as the mean RT is subtracted in the first step of its calculation. However, dividing the same raw RT cost by a longer baseline RT in older adults results in an apparent advantage for older adults in the proportional RT costs in some cases.

Though we simulated data from plausible parameter ranges, we caveat the interpretation of the absolute Type I error rates in that they are dependent on the “noise” produced by variation in the other parameters. For example, if drift rates and boundary separation were held constant across all individuals, variation in scores would be driven only by nondecision time, and would produce a larger false positive rate for the proportional RT costs in Scenario D. Though it is unlikely that such variability would be absent, the magnitude of it may vary with tasks and samples. The broad pattern of results is not specific to the DDM; see online supplementary material for simulations with another common decision model, the linear ballistic accumulator (LBA) model.

## The *z* Transformation and General Slowing

**Figure 3 fig3:**
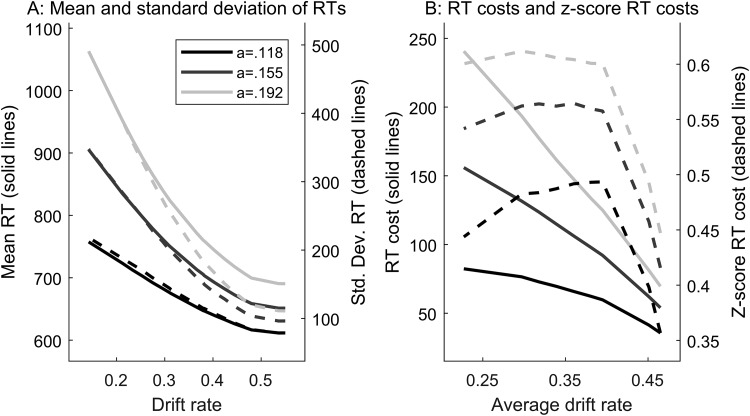
(A) The relationship between the mean response time (RT; left *y*-axis; solid lines) and *SD* of RTs (right *y*-axis; dashed lines) simulated from the diffusion model at varying levels of boundary separation (a; different color lines) and drift rates (*x*-axis). There is a nonlinear relationship between drift rate and both the mean and *SD* of RTs. However, the relationship between the mean and *SD* themselves is approximately linear (see supplementary material D). (B) The relationship between average drift rates and both RT costs (solid lines) and *z*-score costs (dashed lines). Average drift rates refer to the average from easy and hard conditions, with a difference between conditions of .17. On the right side of the plot it can be seen that there is a sharp change in the *z*-score cost at high average drift rates. This occurs because a change in drift rate has relatively little effect on behavior in the easy condition at high values. See main text and supplementary material D for details.

Our observation that the *z*-score RT cost does not fully control the Type I error rate in the general slowing scenario conflicts with the findings of Faust et al. (1999). It also may appear counterintuitive given the observation that the DDM produces an approximately linear relationship between the mean and *SD* of RTs with changes in drift rate (Wagenmakers & Brown, 2007; Wagenmakers, Grasman, & Molenaar, 2005). To understand this discrepancy, we conducted an additional simulation based on the parameter ranges used in our general slowing scenario. We simulated data for a single individual at each parameter combination, with 500,000 trials each in easy and hard conditions. As in our general slowing scenario, we varied the drift rates for both easy and hard conditions while keeping the difference between conditions fixed at .17. In Figure 3A, we plot the relationship between drift rate (*x*-axis) and the mean RT (left *y*-axis) and *SD* of RTs (right *y*-axis) for three levels of boundary separation. In Figure 3B we plot the relationship between drift rates (now averaged over easy and hard conditions) and both the raw RT cost and *z*-score cost. See supplementary material D for additional information.[Fig-anchor fig3]

First, note in Figure 3A that the mean (solid lines) and *SD* (dashed lines) of RTs change at different rates depending on both drift rate and boundary separation (see also Ratcliff et al., 2000). In our general slowing simulations, we used mean drift rates of .48 and .466 (*SD* = .07) for the easy condition in our younger and older (*d* = .02) adults, respectively. Examining the bottom right corner of Figure 3A, the slopes are relatively shallow in this range, indicating little change in the mean and *SD* of RTs at high drift rates. In contrast, the slopes are relatively steep in the range of drift rates used for the hard condition (.31 and .296 for younger and older adults, respectively). The result of this is that some older adults would produce similar RTs to young adults in the easy condition but produce relatively slower and more variable RTs in the hard condition. The *z*-score transformation does not correct for this, as its intended aim is to correct for slower RTs in *both* conditions and leave the within-subject effect intact. This behavior can be clearly seen in the right side of Figure 3B, where the *z*-score costs (dashed lines) show a steep change at higher average drift rates. At lower average drift rates, where behavior in the easy condition is also be affected, the *z*-score cost shows better control for general slowing.

Note that though drift rates in the range of .5 are at the high end of what is typically observed in fits to empirical data, they are based on previous aging studies (Ratcliff, Thapar, et al., 2004; see also Ratcliff, Thapar, & McKoon, 2006b). The patterns we observe in Figure 3 are also consistent with the observation that the DDM produces an approximately linear relationship between the mean and *SD* of RTs (e.g., Wagenmakers & Brown, 2007). In Figure 3A, it can be seen that one generally increases with the other (see also supplementary material D). However, the relationship between the model parameters and the simulated behavior is nonlinear.

## Discussion

To summarize, if we use accumulation models as a reference framework, none of the raw or transformed behavioral measures uniquely identifies domain specific deficits. The *z*-score cost showed lower Type I error rates than both the raw and proportional RT costs in the scenarios of general slowing (B) and strategic slowing (C), though they still notably exceeded the nominal rate (15.5 and 61.5%, respectively, at the largest effect sizes). The *z*-score costs were unaffected by changes in nondecision time (D). Proportional RT costs show relatively little advantage over raw RT costs, and group differences in processing could be reduced or even reversed by differences in nondecision time.

Transformed RT costs have been used prominently in the aging literature to examine whether older adults show deficits in specific cognitive mechanisms in the presence of general slowing (e.g., Colcombe et al., 2005; Gold et al., 2013; Gratton et al., 2009; Henry et al., 2015; Lawo & Koch, 2014; Truong & Yang, 2014; Yang & Hasher, 2007; Zhu et al., 2010). Researchers in a given cognitive domain may wish to remain neutral with respect to quantitative models of choice RT per se, however, an underlying quantitative relationship is implicitly assumed by these transformations. Examining the relationship between the transformed costs and a widely used framework of choice RT allows us to critically evaluate the different scaling assumptions made by the transformations, as well as identify where conclusions may converge or diverge between the two approaches. The results of our simulations indicate that using the *z*-score transformation is preferable to using raw RT costs or proportional costs, as recommended by Faust et al. (1999). However, *z*-score costs still show increased Type I error rates in our scenarios of general slowing and strategic slowing.

### Plausibility of Scenarios

The scenarios that we describe are not atypical—increases in boundary separation and nondecision time in older adults have been reported in numerous studies that have applied the DDM, and similar explanations have been suggested outside of the context of a specific model (Basowitz & Korchin, 1957; McKoon & Ratcliff, 2013; Ratcliff & McKoon, 2015; Ratcliff, Thapar, et al., 2004; Ratcliff et al., 2006a, 2006b, 2011; Schuch, 2016; Spaniol et al., 2006; Starns & Ratcliff, 2010; Strayer & Kramer, 1994; Thapar et al., 2003). The extent to which these factors fully account for observed slowing in older adults is the subject of some debate (Myerson, Adams, et al., 2003; Verhaeghen, 2014), though their presence in some form is less controversial. A detailed evaluation of the evidence for general slowing, and for domain-specific deficits, can be seen in these and other reviews (e.g., Verhaeghen, 2011). Here, we focus on the interpretation of the metrics themselves.

Our simulations show that the rates of Type I errors in the behavioral costs are dependent on the size of the effect in the underlying parameters. Notably, the upper end of effect sizes we simulated (*d* = 1.4) exceeds the level traditionally considered to be a “large” effect (*d* = 0.8). A consideration then is the extent to which the confounding effects (Scenarios B–D) are plausibly large enough in real samples that they are likely to contaminate traditionally used measures. We can evaluate this by examining previously reported fits of the DDM to younger and older adult data across multiple tasks (Ratcliff et al., 2006a, their Table 3; Ratcliff, Thapar, & McKoon, 2010, their Tables 2 and 3). Ratcliff et al. (2006a) tested young adults, 60–74 year olds, and 75–85 year olds on numerosity discrimination, letter discrimination, brightness discrimination, and recognition memory tasks. Ratcliff et al. (2010) used numerosity discrimination, lexical decision, and recognition memory tasks with young adults, 60–74, and 75–90 year olds. We calculated the effect size for each parameter/condition in the two articles, and report the average effect sizes for each parameter in Table 4. The average effect sizes for boundary separation and nondecision time are in the upper range of, or they exceed, those used in our simulations. For drift rates, group differences are smaller and inconsistent, with older adults sometimes showing higher values (better performance) in individual tasks.[Table-anchor tbl4]

We focus on the consequences for the interpretation of *z*-score costs, as these showed the lowest false positive rates in our simulations. We could infer from Table 4 that differences in average drift rates are less likely to be problematic because they tend not to be large. This is not true across all domains, however. For example, Ratcliff, Thapar, and McKoon (2011) show small and large age related declines in drift rates for item recognition and associative recognition, respectively. The large age differences commonly observed in boundary separation are potentially more problematic for interpretations of the *z*-score cost.

Though we simulated the effects of changing each parameter in isolation here, we emphasize that individuals and groups may vary on multiple underlying dimensions. This is not to say that the scenarios we outline are not dissociable, as drift rates, boundary separation and nondecision time typically show low or inconsistent correlations between each other (Ratcliff & McKoon, 2015; Ratcliff et al., 2010, 2011). Nevertheless, some combinations of the scenarios we outline could be particularly problematic for interpreting the underlying source(s) of slowing. In supplementary material C, we examine illustrative cases where older adults differ from young adults in both strategic slowing and either a domain specific deficit or general slowing. This makes the data patterns difficult to interpret, as strategic slowing increases group differences in RT costs while having the opposite effect on error costs.

### Relation to Previous Work

We are not the first to question the utility of proportional RT costs, or other methods for controlling for confounding factors when examining processing speed in aging. Faust and colleagues (Faust et al., 1999) evaluated both proportional RT costs and *z*-scores in the context of their rate-amount model, which predicts individuals’ RTs in a given condition on the basis of a relation between the amount of processing required in a condition and the individual’s processing speed. Faust et al. note that a conceptual similarity between their model and the accumulation of evidence to a boundary in models such as the DDM. However, unlike the DDM, the rate and amount model is a model of behavior at the group level, in that it describes the relationship between an individual’s RTs in one condition to their average, and to that of others in the group. When the assumptions of their model were met, Faust et al. show that *z*-scores are an appropriate transformation to control for processing speed differences. In our simulations, the *z*-score transformation reduced (but did not eliminate) the rate of false positives in the general slowing and strategic slowing scenarios, with the latter producing larger effects. The observation that the *z*-score transformation does not control for differences in boundary separation is not at odds with Faust et al.’s (1999) conclusions, in that they assume that variation in the amount of processing required within a task is determined by the difficulty of the condition, not individual differences in strategy.

Regarding general slowing, the discrepancy between our results and Faust et al.’s (1999) may reflect the different assumptions and approaches to data generation Faust et al. simulated data by sampling means and *SD*s of RTs in accordance with the relationship predicted by the rate-amount model. In other words, “slowing” was implemented as a change in behavior. In contrast, we implemented slowing as a change in drift rate; a model parameter theorized to represent the efficiency of the underlying processing. Critically, a change in the latent model parameter does not always correspond to an equivalent change in behavior. In our simulations, a decrease in drift rates in both conditions in a hypothetical older adult relative to a younger adult could manifest in behavior only in the more difficult condition. In a situation where data are produced by a diffusion process and the parameters fall within a certain range, the *z*-score transformation may provide better control over Type I error rates than we observe (note that Faust et al. make additional assumptions about the group level structure of the data that we do not make here; see also Leite, Ratcliff, & White, 2007; Myerson, Hale, Zheng, Jenkins, & Widaman, 2003; Ratcliff et al., 2000). However, the previous data on which we based our simulations (Ratcliff, Thapar, et al., 2004; see also Ratcliff et al., 2006a) suggest that ranges may go beyond those where Type I errors are kept below the nominal rate.

We emphasize that it is not our position that any single analytical approach or model is correct; we do not *know* the generating model for data from human participants. Rather, by illustrating where conclusions drawn from one approach may not be robust to another analytical approach or theoretical perspective, our aim is to highlight the value of triangulating a range of approaches within and between studies (Munafò & Davey Smith, 2018; Salthouse & Hedden, 2002).

### Recommendations

Theorists have previously recommended the use of RT transformations, in particular the *z*-score, on the basis that they provide greater control over Type I error rates when used in conjunction with the analysis of raw RTs (Faust et al., 1999). Our findings do not contradict this advice; we observed lower Type I error rates when using the *z*-score relative to examining raw RT costs. However, our observation of elevated (>5%) Type I error rates when using the *z*-score in some scenarios is a reminder that researchers should also seek convergence from other methods, such as those that incorporate accuracy (Ratcliff et al., 2000; Salthouse & Hedden, 2002). This is not to suggest that every study should conform to a particular design that allows for a range of analytical methods to be applied. We focus on approaches that incorporate accuracy because they are easily applied to many existing tasks, and because of the broad literature that links *ability* in a given cognitive domain to both speed and accuracy (that is not limited to sequential sampling models; Pachella, 1974; Salthouse & Hedden, 2002; Wickelgren, 1977).

It naturally follows from our framing of different hypothetical sources of slowing in the context of the DDM that fitting the model itself is one such method that could be used to supplement analyses. In particular, freely available software packages are available to fit a hierarchical Bayesian implementation of the model (Wiecki, Sofer, & Frank, 2013). Hierarchical methods assume that individuals are sampled from one or more populations, and simultaneously estimate parameters at the group and individual level. This is a benefit where the number of trials per subject is relatively low, as may be the case in aging research, as the group level information can inform the individual estimates. There are other software packages available (Vandekerckhove & Tuerlinckx, 2008; Voss & Voss, 2007; Wagenmakers, Van Der Mass, & Grasman, 2007), and other choice RT models available (e.g., the LBA; Brown & Heathcote, 2008). Our simulations using the LBA in supplementary material B produce similar results to those in the main text, and conclusions about psychological processes are generally thought to not depend on the choice of model (Donkin et al., 2011; Ratcliff et al., 2005).

Though not a substitute for quantitative analysis, our simulations also point toward heuristics that can be used to identify confounds. For example, if older adults show increased RT costs and *z*-score costs relative to younger adults, but decreased or similar error costs, then this would point toward an influence of strategic slowing. A general slowing scenario could lead to lower accuracy in the baseline condition, though this may be particularly difficult to detect in real data (see below). We make the assumption here that RT, or processing speed, and accuracy are not independent. This view is not dependent on the framework of sequential sampling models (cf. Salthouse & Hedden, 2002; Wickelgren, 1977), though an advantage of the models is that the relationship is specified. Many alternative methods entail the separate analysis of RTs and accuracy, which leaves the researcher to gauge the relative importance of an effect (or the absence of one) in each in a given dataset (Salthouse & Hedden, 2002).

However, there may be tasks or data for which a model such as the DDM is not applicable, or researchers may simply not wish to commit to an interpretation within a specific framework. Salthouse and Hedden (2002) discuss a variety of approaches that can be used to examine the consistency of interpretations, for example, the use of composite scores (cf. Vandierendonck, 2017), the generation of speed–accuracy trade-off functions, and the use of response deadlines tasks. A notable consideration for all of these techniques is that it is often the intention of researchers (or participants) to avoid large numbers of errors in performance. In the context of the DDM, this could be seen as participants adopting a level of response caution that minimizes errors irrespective of their drift rates. This has the consequence of making within-subject effects in accuracy difficult to detect, while producing large RT effects, as seen in Scenario C (see also Ratcliff et al., 2000; Wickelgren, 1977). This is difficult to address solely through analysis methods, and it is also difficult to fit choice RT models to data where no errors are made. Flawless accuracy in all conditions may reflect a relatively extreme scenario, however. As noted, errors are not completely absent in data sets where RT transformations have previously been considered (Hale et al., 1995).

Finally, choice RT tasks often consist of multiple conditions and/or multiple response options, whereas we focus on binary choice performance in two conditions here. Most of the issues we discuss extend to more complex tasks, and analysis methods can be extended to accommodate them. Extensions of the different choice RT models been proposed that accommodate tasks with multiple response options (for an overview, see Tsetsos, Usher, & McClelland, 2011). In the case of accumulator models such as the LBA, each response option is simply assigned a unique accumulator, so hypothetically there is no constraint on the number of response options that can be modeled. Alternatively, in cases where no systematic difference between response options is expected, some theorists have suggested that the regular DDM could be fit to data where responses are collapsed to be coded simply as correct or incorrect (Voss, Nagler, & Lerche, 2013). The same concerns about RT scaling effects, and the value of incorporating accuracy into analyses, carry across to these extensions.

To conclude, there is understandable appeal of easy-to-calculate metrics for studying group differences in RTs, however, theorists have emphasized caution in applying these and other methods blindly (Faust et al., 1999; Ratcliff et al., 2000; Ratcliff, Spieler, & McKoon, 2004; Verhaeghen, 2014). A specific relationship between RT costs and overall response speed is (often implicitly) assumed by different transformations, and quantitative models of choice RT provide a useful reference for those scaling assumptions. We recommend against the use of proportional RT costs. The *z*-score costs provide improved control over Type I errors relative to the analysis of raw RTs, though it is sensitive to confounds, and should ideally be interpreted in conjunction with analyses of errors where possible.

## Supplementary Material

10.1037/pag0000298.supp

## Figures and Tables

**Table 1 tbl1:** Parameters Used to Simulate Data from Drift-Diffusion Model, Derived from Ratcliff, Thapar, et al. (2004)

Scenario	Drift rate easy (v1)	Drift rate hard (v2)	Boundary separation (a)	Nondecision time (Ter)
Domain-specific deficit (A)	.480	**Young: .310**	.155	490
		**Old: .301–.248**		
General slowing (B)	**Young: .480**	**Young: .310**	.155	490
	**Old: .466–.382**	**Old: .296–.212**		
Strategic slowing (C)	.480	.310	**Young: .127**	490
			**Old: .134–.179**	
Nondecision time (D)	.480	.310	.155	**Young: 440**
				**Old: 450–510**
*SD*s (all scenarios)	.07	.07	.037	50
*Note.* Individual parameter values in each scenario were generated from a normal distribution with means given in the first four rows and the *SD*s shown in the bottom row. Mean parameters that were varied between groups in each scenario are highlighted in bold, with the range shown for older adults. In Scenarios B, C, and D, the effect between groups in each scenario can be calculated by multiplying the Cohen’s *d* value by the *SD* (e.g., .2 × 50 ms for the smallest effect in nondecision time in Scenario D). For Scenario A, the difference of interest is the group difference in the difference between easy and hard drift rates. The *SD* of the difference (easy–hard drift rates) was .044.				

**Table 2 tbl2:** Mean Reaction Times and Error Rates for Simulated Young and Old Adults

Scenario	Young		Old (*d* = .2)		Old (*d* = 1.4)	
	Easy	Hard	Easy	Hard	Easy	Hard
Reaction time (ms)						
A: Domain	667 (69)	755 (106)	665 (69)	760 (108)	668 (67)	806 (122)
B: General	667 (71)	755 (108)	670 (72)	767 (111)	705 (90)	829 (139)
C: Strategic	635 (70)	700 (99)	641 (70)	711 (102)	696 (73)	804 (114)
D: Nondecision	617 (71)	704 (107)	629 (71)	719 (109)	688 (72)	779 (112)
Error rates (%)						
A: Domain	1 (1)	4 (4)	1 (1)	4 (4)	1 (1)	8 (6)
B: General	1 (1)	4 (4)	1 (1)	5 (5)	2 (2)	11 (7)
C: Strategic	2 (3)	6 (6)	1 (2)	5 (5)	0 (1)	3 (3)
D: Nondecision	1 (2)	4 (4)	1 (1)	4 (4)	1 (1)	4 (4)
*Note.* *SD*s given in parentheses. Older adult means are reported for the smallest (*d* = .2) and largest (*d* = 1.4) effect sizes simulated. Means for young adults come from the *d* = .2 scenarios, though only the parameter distributions used to simulate older adult data varied across effect sizes.						

**Table 3 tbl3:** Percentage of Significant (p < .05) t-Tests from 5,000 Simulated Experiments

Scenario	Effect size	Mean RT	RT cost	Proportional cost	*z*-score cost	Mean error	Error cost
A: Domain specific deficit	.2	2.8 | 2.1	6.8 | .8	7.9 | .6	10.2 | .2	3.3 | 1.4	5.2 | .9
	.5	3.5 | 1.9	16.5 | .2	20 | .2	24.9 | 0	11.1 | .1	17.2 | .1
	.8	9.1 | .3	46.3 | 0	52 | 0	70.3 | 0	27.8 | 0	46.9 | 0
	1.1	11.5 | .4	64 | 0	71.6 | 0	89.9 | 0	38.6 | 0	64.2 | 0
	1.4	19.1 | .1	81 | 0	87 | 0	97.2 | 0	58 | 0	83.2 | 0
B: General slowing	.2	4.6 | 1.3	8.3 | .5	8.7 | .4	7.9 | .5	11.2 | .3	12.9 | .2
	.5	6.9 | .7	12.8 | .2	12 | .2	8 | .4	23.3 | .1	27.5 | .1
	.8	22.8 | .1	29.8 | 0	27.6 | .1	10.6 | .4	58.6 | 0	65.6 | 0
	1.1	54 | 0	54.7 | 0	47.2 | 0	16.2 | .2	80.1 | 0	86.5 | 0
	1.4	59.8 | 0	60.9 | 0	52.9 | 0	15.5 | .2	96.2 | 0	98.9 | 0
C: Strategic slowing	.2	5.3 | .8	5 | .6	4.7 | .8	3.4 | 1.6	**.7 | 5.9**	**.9 | 6.1**
	.5	16.3 | .2	15.8 | .1	14.4 | .2	7.8 | .6	**.1 | 18.2**	**.1 | 13.1**
	.8	52.3 | 0	44.6 | 0	40.9 | 0	26.1 | 0	**0 | 41.2**	**0 | 24**
	1.1	87.8 | 0	74.1 | 0	65.1 | 0	35.7 | 0	**0 | 62.1**	**0 | 37**
	1.4	95.6 | 0	87.5 | 0	81.4 | 0	61.5 | 0	**0 | 83.9**	**0 | 60.2**
D: Nondecision time	.2	8.5 | .6	3.5 | 1.7	**2.6 | 2.1**	3.6 | 1.5	2.3 | 2.2	2.8 | 2.2
	.5	15.7 | .2	1.9 | 2.5	**1.1 | 4.7**	2 | 2.8	1.4 | 3.3	1.3 | 3.6
	.8	43.5 | 0	3.3 | 1.9	**1.1 | 4.4**	3.2 | 2	2.6 | 2.1	3 | 1.7
	1.1	68.1 | 0	2.6 | 1.7	**.6 | 6.7**	2.9 | 1.8	3.2 | 1.7	4.2 | 1.5
	1.4	88.3 | 0	3.4 | 1.3	**.6 | 7.7**	2.8 | 2.1	2.6 | 1.8	3 | 1.9
*Note*. Values to the left of the vertical bars are the percentage of pseudo-experiments in which older adults showed significantly slower RTs or larger costs (i.e. worse performance). Values to the right of the vertical bars are the percentage of experiments in which younger adults showed significantly slower RTs or larger costs. Values in bold highlight cases in which simulated older adults typically produced relatively lower costs or lower error rates. If the transformed costs control the Type 1 error rate, then the total proportion of significant effects should be approximately 5% in Scenarios B–D.							

**Table 4 tbl4:** Average Effect Sizes (Cohen’s d) for Group Differences in Four Tasks Reported in Ratcliff et al. (2006a) and Three Tasks in Ratcliff et al. (2010)

Parameter	Young adults vs. 60–74 year olds	Young adults vs. 75–90 year olds
Drift rate (v)	−.00	−.26
Boundary separation (a)	.98	1.55
Nondecision time (Ter)	1.73	1.81
*Note*. Effect sizes for each parameter are averaged across studies, tasks and conditions. Positive effect sizes reflect higher values in older adults.
