# Supplementary material for: The Mapping Between Transformed Reaction Time Costs and Models of Processing in Aging and Cognition
Source: Psychol Aging. 2018 Oct 8;33(7):1093–104. doi: 10.1037/pag0000298 (PMC6233521; doi:10.1037/pag0000298)
Supplement: Supplementary file 1 [file PAG-2017-1085Suppl.zip › PsychAging2ndRevSupp.docx]

**Supplementary Material A**

This section contains the descriptive statistics (means and standard deviations) for the data used in the simulations in the main text.

Table A1.

*Means and standard deviations (in parentheses) for data simulated from the drift diffusion model. Parameters used for simulations are reported in the main text (Table 1). Scenarios reflect changes in: A) a domain specific deficit, B) general slowing, C) strategic slowing, D) non-decision time.*

|  | d=0.2 | | | | | | | | | | | |
| --- | --- | --- | --- | --- | --- | --- | --- | --- | --- | --- | --- | --- |
|  | Younger | | | | | | Older | | | | | |
| Scenario | Easy RT | Hard RT | Easy % error | Hard % error | P. cost | Z cost | Easy RT | Hard RT | Easy % error | Hard % error | P. cost | Z cost |
| A | 667 (69) | 755 (106) | 1 (1) | 4 (4) | 13 (7) | 0.49 (0.15) | 665 (69) | 760 (108) | 1 (1) | 4 (4) | 14 (8) | 0.51 (0.15) |
| B | 667 (71) | 755 (108) | 1 (1) | 4 (4) | 13 (7) | 0.49 (0.15) | 670 (72) | 767 (111) | 1 (1) | 5 (5) | 14 (7) | 0.51 (0.15) |
| C | 635 (70) | 700 (99) | 2 (3) | 6 (6) | 10 (6) | 0.44 (0.15) | 641 (70) | 711 (102) | 1 (2) | 5 (5) | 11 (6) | 0.45 (0.15) |
| D | 617 (71) | 704 (107) | 1 (2) | 4 (4) | 14 (8) | 0.48 (0.15) | 629 (71) | 719 (109) | 1 (1) | 4 (4) | 14 (8) | 0.49 (0.15) |
|  | d=0.5 | | | | | | | | | | | |
|  | Younger | | | | | | Older | | | | | |
| Scenario | Easy RT | Hard RT | Easy % error | Hard % error | P. cost | Z cost | Easy RT | Hard RT | Easy % error | Hard % error | P. cost | Z cost |
| A | 669 (71) | 759 (108) | 1 (1) | 4 (4) | 13 (7) | 0.49 (0.15) | 666 (71) | 770 (115) | 1 (1) | 5 (5) | 15 (8) | 0.54 (0.15) |
| B | 671 (72) | 760 (106) | 1 (1) | 4 (4) | 13 (7) | 0.49 (0.15) | 677 (78) | 779 (118) | 1 (2) | 6 (5) | 15 (7) | 0.51 (0.14) |
| C | 635 (70) | 701 (100) | 2 (3) | 6 (5) | 10 (6) | 0.44 (0.15) | 651 (72) | 728 (106) | 1 (2) | 5 (5) | 12 (7) | 0.46 (0.15) |
| D | 618 (71) | 706 (109) | 1 (1) | 4 (4) | 14 (8) | 0.49 (0.15) | 640 (71) | 728 (108) | 1 (1) | 4 (4) | 13 (7) | 0.48 (0.15) |
|  | d=0.8 | | | | | | | | | | | |
|  | Younger | | | | | | Older | | | | | |
| Scenario | Easy RT | Hard RT | Easy % error | Hard % error | P. cost | Z cost | Easy RT | Hard RT | Easy % error | Hard % error | P. cost | Z cost |
| A | 667 (71) | 754 (106) | 1 (1) | 4 (4) | 13 (7) | 0.48 (0.15) | 667 (72) | 783 (119) | 1 (1) | 6 (5) | 17 (9) | 0.58 (0.14) |
| B | 668 (71) | 757 (108) | 1 (1) | 4 (4) | 13 (7) | 0.49 (0.15) | 688 (81) | 799 (126) | 1 (2) | 7 (6) | 16 (8) | 0.52 (0.14) |
| C | 635 (70) | 701 (103) | 2 (3) | 6 (6) | 10 (6) | 0.44 (0.15) | 669 (72) | 759 (109) | 1 (1) | 4 (4) | 13 (7) | 0.49 (0.15) |
| D | 617 (71) | 705 (108) | 1 (1) | 4 (4) | 14 (8) | 0.49 (0.15) | 658 (71) | 748 (108) | 1 (1) | 4 (4) | 13 (7) | 0.49 (0.15) |
|  | d=1.1 | | | | | | | | | | | |
|  | Younger | | | | | | Older | | | | | |
| Scenario | Easy RT | Hard RT | Easy % error | Hard % error | P. cost | Z cost | Easy RT | Hard RT | Easy % error | Hard % error | P. cost | Z cost |
| A | 667 (72) | 756 (108) | 1 (1) | 4 (4) | 13 (7) | 0.49 (0.15) | 667 (69) | 792 (120) | 1 (1) | 7 (6) | 18 (9) | 0.61 (0.14) |
| B | 664 (70) | 753 (107) | 1 (1) | 4 (4) | 13 (7) | 0.49 (0.15) | 699 (84) | 821 (133) | 1 (2) | 8 (7) | 17 (8) | 0.53 (0.14) |
| C | 631 (69) | 696 (98) | 2 (3) | 6 (5) | 10 (6) | 0.44 (0.15) | 683 (71) | 782 (109) | 0 (1) | 3 (4) | 14 (7) | 0.5 (0.15) |
| D | 618 (70) | 708 (109) | 1 (1) | 4 (4) | 14 (8) | 0.49 (0.15) | 673 (70) | 763 (107) | 1 (1) | 4 (4) | 13 (7) | 0.49 (0.15) |
|  | d=1.4 | | | | | | | | | | | |
|  | Younger | | | | | | Older | | | | | |
| Scenario | Easy RT | Hard RT | Easy % error | Hard % error | P. cost | Z cost | Easy RT | Hard RT | Easy % error | Hard % error | P. cost | Z cost |
| A | 665 (70) | 756 (109) | 1 (2) | 4 (4) | 13 (7) | 0.49 (0.16) | 668 (67) | 806 (122) | 1 (1) | 8 (6) | 20 (10) | 0.64 (0.14) |
| B | 665 (71) | 754 (110) | 1 (2) | 4 (4) | 13 (7) | 0.49 (0.15) | 705 (90) | 829 (139) | 2 (2) | 11 (7) | 17 (8) | 0.52 (0.14) |
| C | 634 (70) | 699 (102) | 2 (3) | 6 (6) | 10 (6) | 0.43 (0.15) | 696 (73) | 804 (114) | 0 (1) | 3 (3) | 15 (8) | 0.52 (0.15) |
| D | 616 (73) | 703 (108) | 1 (2) | 4 (4) | 14 (7) | 0.49 (0.15) | 688 (72) | 779 (112) | 1 (1) | 4 (4) | 13 (7) | 0.49 (0.15) |

**Supplementary Material B**

**Linear ballistic accumulator simulations**

In the main text, we simulate data from the drift-diffusion model (DDM) under four scenarios that could produce slower reaction times (RT) in one group relative to another. Here, we conduct analogous simulations using an alternative model; the Linear Ballistic Accumulator (LBA; Brown & Heathcote, 2008). As with the DDM, the LBA assumes that evidence is accumulated to a threshold (b) over time. However, while accumulation in the DDM is subject to random noises, the accumulation is linear in the LBA. Further, in the LBA the evidence for the correct and incorrect responses are represented by separate accumulators. The mean drift rate (v, or vc) refers to the drift rate for the correct response, while the drift rate for the incorrect response (ve) is typically set to 1-vc (though see Donkin, Brown, & Heathcote, 2009). Drift rates are drawn from normal distributions with a common standard deviation (s). Start points for the accumulation process are randomly drawn from a uniform distribution ranging from 0 to A. As with the DDM, a non-decision time constant (Ter) is added to the RT for each trial.

Despite the different model architectures, the scenarios we presented in the context of the DDM translate easily to the LBA (see Figure S1). To implement a domain specific deficit (Scenario A), as with the DDM, we kept the drift rates for easy trials the same for younger and older adults, and made the drift rates for hard trials relatively smaller for older adults. For general slowing (Scenario B), we reduced the drift rates for both easy and hard trials in older adults. For the strategic slowing scenario (Scenario C), we increased the height the boundary (B), while keeping the edge of the start point distribution (A) constant. Finally, we increased the non-decision time constant in older adults for Scenario D.


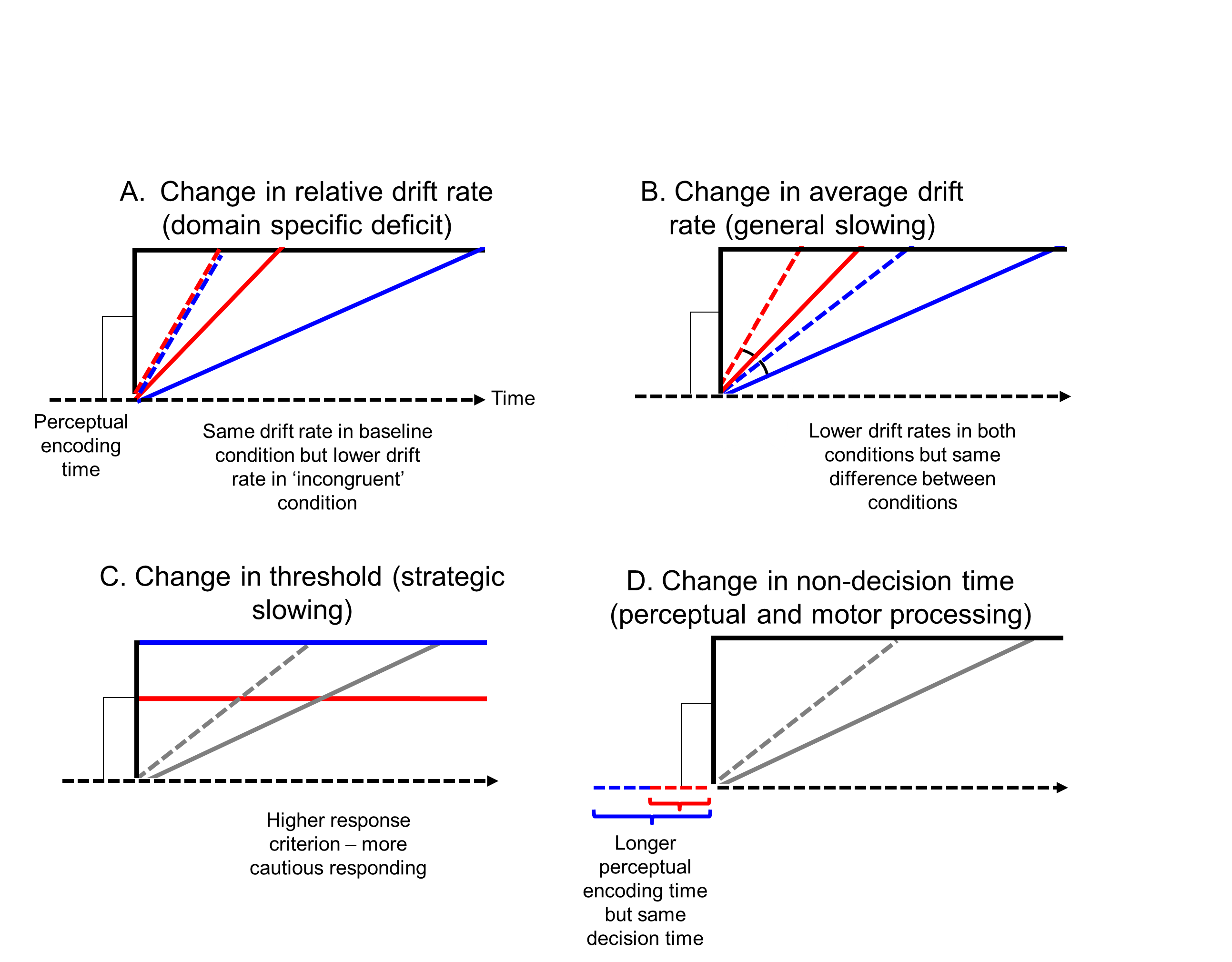


*Figure B1.* Four scenarios in which two individuals could produce different reaction times in the Linear Ballistic Accumulator framework. In all cases, the individual that would produce slower RTs is portrayed in blue, and the faster individual is shown in red. *A*. In this scenario, both individuals produce the same drift rates in the baseline condition (red and blue dashed lines), but one shows a domain specific deficit, in which the drift rate in the more difficult condition is lower. *B.* Global slowing, reflecting lower mean drift rates in both conditions while maintaining the same difference between drift rates in both fast and slow individuals. *C*. Strategic slowing (threshold). The individual represented by the red line requires less evidence in order to make a response, resulting in faster RTs (and more errors). In the LBA, caution is more appropriately characterised the relative distance between the threshold and the mean of the uniform start point distribution (b-A/2). In these simulations, we hold the parameter A constant, so caution is implemented as a change in threshold only. *D*. A change in non-decision time. Drift rates and thresholds are unchanged.

In order to provide comparable results to our simulations in the main text, we derived parameters for our LBA simulations by first fitting the LBA to data generated from the DDM. For this, we generated 200 each younger and older adults using our reference parameters for each group (see Table 1 in the main text). We then fitted the LBA to these data using freely available R code (Donkin, Averell, Brown, & Heathcote, 2009). We then calculated 5% trimmed means and standard deviations for each parameter, and used these to calculate effect sizes for each scenario analogously to the simulations in the main text. Two adjustments were made to the parameters obtained from our fits, however. First, the LBA fits gave a relatively large standard deviation for the drift rates within subjects (s=.475). This is likely a consequence of accuracy being very high in the simulated data, entailing high average drift rates. In order to prevent large effect size scenarios producing poor accuracy, we used a lower value for the s parameter (.15), which is within the range of previous simulations (Donkin et al., 2011). The second change was to fix the parameter A, which represents the size of the uniform distribution of starting points. The reason for this is that, while response caution in the DDM is typically characterised as boundary separation, the analogous quantity in the LBA is the relative difference between threshold (B) and the edge of the starting point distribution (A). For simplicity, we fixed A and varied B.

Table B1 shows the parameters used for the LBA simulations.

Table B1.

*Parameters used to simulate data from Linear Ballistic Accumulator. Parameters varied in each scenario are shown in bold, all other parameters were held constant. Standard deviations of varied parameters are shown in parenthesis.*

| Scenario | Drift rate easy (v1) | Drift rate hard (v2) | Boundary (B) | Non-decision time (Ter) |
| --- | --- | --- | --- | --- |
| Domain-specific deficit (A) | **1.48** | **Young:** **1.07**  **Old: 1.051 - .9372** | .410 | .440 |
| General slowing (B) | **Young: 1.48**  **Old: 1.45 – 1.27** | **Young: 1.07**  **Old: 1.04 - .86** | . 410 | .440 |
| Strategic slowing (C) | 1.48 | 1.07 | **Young: .33**  **Old: .343 - 421** | .440 |
| Non-decision time (D) | 1.48 | 1.07 | . 410 | **Young: .405**  **Old: .416 - .482** |
| Standard deviation | .15 | .15 | .065 | .055 |
| Note. The parameter describing the edge of the start point distribution, A, was fixed at .235 for all simulations. Within-subject variation in drift rates (s) was fixed to .15. The mean drift rate for the error accumulator was fixed to one minus the mean drift rate of the correct response. | | | | |

As with our DDM simulations in the main text, we created pools of 2000 younger and older adults in each effect size for each scenario. We then randomly sampled from the pools to create 5000 pseudo-experiments with N=30 in each group.

The calculation of mean RTs and RT costs excluded incorrect responses. Trials with decision times longer than 3 seconds were removed for RT analyses.

A plot of the effect sizes produced in each measure in relation to the parameter manipulations s shown in Figure B2, while Table B2 shows the percentage of pseudo-experiments in which the group difference was significant in an independent t-test (p<.05). Note, though we aimed to produce approximately similar data ranges in our LBA and DDM scenarios, we do not intend for Table B2 to be directly compared to Table 2 in the main text, though the quantitative results are relatively similar. As drift rates remained relatively high, even in large effect size scenarios, errors were less frequent than in our DDM simulations.

Figure B2. Relationship between the effect size in LBA manipulated in each scenario (x-axis) and the effect size observed in the behavioural measures derived from the simulated data (y-axis). Positive effect sizes on the y-axis indicate larger costs in the older adult group. See Table B1 and Figure B1 for parameters manipulated in each scenario.

Table B2.

*Percentage of significant (p<.05) t-tests from 5000 simulated experiments. Values to the left of the vertical bar are the percentage in which older adults showed higher costs (worse performance), values to the right of the vertical bar are those in which younger adults showed higher costs.*

| Scenario | Effect size | Mean RT | RT cost | Proportional Cost | Z-score cost | Mean error | Error cost |
| --- | --- | --- | --- | --- | --- | --- | --- |
| A: Domain specific deficit | 0.2 | 3.9 \| 1.3 | 6.8 \| 0.7 | 7.1 \| 0.4 | 6.6 \| 0.6 | 1.2 \| 0.4 | 1.2 \| 0.4 |
|  | 0.5 | 4.5 \| 1.1 | 25 \| 0.1 | 29.1 \| 0.1 | 27.9 \| 0.1 | 3.6 \| 0.2 | 3.6 \| 0.2 |
|  | 0.8 | 9.2 \| 0.4 | 64.1 \| 0 | 71.4 \| 0 | 69.1 \| 0 | 10.9 \| 0 | 10.9 \| 0 |
|  | 1.1 | 10.9 \| 0.4 | 83.8 \| 0 | 89.9 \| 0 | 87.3 \| 0 | 21.9 \| 0 | 22 \| 0 |
|  | 1.4 | 13.8 \| 0.2 | 96.8 \| 0 | 99.2 \| 0 | 98.7 \| 0 | 29.3 \| 0 | 29.4 \| 0 |
| B: General slowing | 0.2 | 4.5 \| 1.1 | 5.9 \| 0.8 | 5.5 \| 1 | 3.1 \| 2 | 2.4 \| 0.2 | 2.4 \| 0.2 |
|  | 0.5 | 8.4 \| 0.4 | 18 \| 0.2 | 18.3 \| 0.2 | 5.5 \| 1 | 10.1 \| 0 | 10.2 \| 0 |
|  | 0.8 | 26.3 \| 0.1 | 47.2 \| 0 | 41.8 \| 0 | 11.7 \| 0.2 | 23.6 \| 0 | 23.5 \| 0 |
|  | 1.1 | 33.6 \| 0 | 67.1 \| 0 | 63.5 \| 0 | 14.7 \| 0.3 | 53.7 \| 0 | 53.7 \| 0 |
|  | 1.4 | 66.9 \| 0 | 92.5 \| 0 | 89.7 \| 0 | 24.6 \| 0 | 84 \| 0 | 84.2 \| 0 |
| C: Strategic slowing | 0.2 | 6.2 \| 0.6 | 8.1 \| 0.4 | 7.2 \| 0.6 | 9.1 \| 0.3 | **0.5 \| 3.3** | **0.5 \| 3.2** |
|  | 0.5 | 27 \| 0 | 23.3 \| 0 | 17 \| 0.1 | 28.2 \| 0 | **0.1 \| 6.6** | **0.1 \| 6.5** |
|  | 0.8 | 49.6 \| 0 | 43.8 \| 0 | 33.8 \| 0 | 58 \| 0 | **0 \| 10.9** | **0 \| 10.8** |
|  | 1.1 | 81.7 \| 0 | 73.6 \| 0 | 57 \| 0 | 85.9 \| 0 | **0 \| 16.8** | **0 \| 16.4** |
|  | 1.4 | 93.9 \| 0 | 82.3 \| 0 | 62.1 \| 0 | 90.6 \| 0 | **0 \| 21.5** | **0 \| 21.3** |
| D: Non-decision time | 0.2 | 8.9 \| 0.6 | 2.8 \| 2.2 | **1.8 \| 3.2** | 2.9 \| 2.4 | 0.6 \| 1 | 0.6 \| 1 |
|  | 0.5 | 23.8 \| 0.1 | 2.4 \| 2.5 | **0.7 \| 7** | 2.5 \| 2.9 | 0.7 \| 0.7 | 0.7 \| 0.8 |
|  | 0.8 | 47.4 \| 0 | 3.1 \| 2 | **0.5 \| 7.7** | 2.6 \| 1.9 | 0.7 \| 0.7 | 0.7 \| 0.7 |
|  | 1.1 | 73.5 \| 0 | 2.6 \| 2.1 | **0.3 \| 13** | 2.1 \| 2.8 | 1.1 \| 0.5 | 1 \| 0.5 |
|  | 1.4 | 95.9 \| 0 | 2.6 \| 1.9 | **0.1 \| 21.4** | 2.5 \| 2.1 | 0.7 \| 0.7 | 0.7 \| 0.6 |

The results of the LBA simulations were similar to that reported in the main text for the DDM. The z-score transformation again showed the greatest error control in the general slowing scenario, though unlike in the DDM simulations, it did not perform best with respect to changes in response caution. This is perhaps due to the tendency of the LBA to produce particularly long RTs on some trials, when low drift rates are sampled for both accumulators, which may distort both the mean and variance of RTs.

The descriptive statistics for these data can be seen in Table B3. Note that the z-score costs are somewhat larger in both groups than in the DDM simulations. This is due to reducing the parameter value for the standard deviation of the drift rates as described above, which resulted in lower standard deviations for the RTs. This affected both groups, so does not alter our conclusions about the between group comparisons.

Table B3.

*Descriptive statistics for LBA simulations*

|  | d=0.2 | | | | | | | | | | | |
| --- | --- | --- | --- | --- | --- | --- | --- | --- | --- | --- | --- | --- |
|  | Younger | | | | | | Older | | | | | |
| Scenario | Easy RT | Hard RT | Easy % error | Hard % error | P. cost | Z cost | Easy RT | Hard RT | Easy % error | Hard % error | P. cost | Z cost |
| A | 641 (72) | 724 (92) | 0 (0) | 0 (2) | 13 (5) | 1 (0.21) | 643 (73) | 731 (95) | 0 (0) | 0 (2) | 14 (5) | 1.03 (0.21) |
| B | 643 (72) | 727 (92) | 0 (0) | 0 (1) | 13 (5) | 1 (0.22) | 648 (74) | 736 (96) | 0 (0) | 1 (2) | 14 (5) | 1.01 (0.21) |
| C | 590 (72) | 650 (88) | 0 (0) | 1 (2) | 10 (4) | 0.81 (0.22) | 597 (71) | 660 (87) | 0 (0) | 1 (2) | 11 (4) | 0.85 (0.22) |
| D | 607 (75) | 690 (96) | 0 (0) | 0 (2) | 14 (5) | 0.99 (0.21) | 620 (73) | 703 (94) | 0 (0) | 0 (2) | 13 (5) | 1 (0.21) |
|  | d=0.5 | | | | | | | | | | | |
|  | Younger | | | | | | Older | | | | | |
| Scenario | Easy RT | Hard RT | Easy % error | Hard % error | P. cost | Z cost | Easy RT | Hard RT | Easy % error | Hard % error | P. cost | Z cost |
| A | 641 (72) | 724 (93) | 0 (0) | 0 (1) | 13 (5) | 1 (0.21) | 641 (72) | 737 (97) | 0 (0) | 1 (2) | 15 (6) | 1.07 (0.2) |
| B | 643 (75) | 727 (97) | 0 (0) | 0 (1) | 13 (5) | 1 (0.22) | 652 (76) | 747 (101) | 0 (0) | 1 (4) | 14 (6) | 1.02 (0.21) |
| C | 587 (72) | 648 (89) | 0 (0) | 1 (2) | 10 (4) | 0.82 (0.23) | 611 (72) | 681 (90) | 0 (0) | 0 (1) | 11 (5) | 0.9 (0.22) |
| D | 608 (74) | 692 (95) | 0 (0) | 0 (1) | 14 (5) | 1 (0.21) | 636 (74) | 720 (94) | 0 (0) | 0 (2) | 13 (5) | 1 (0.21) |
|  | d=0.8 | | | | | | | | | | | |
|  | Younger | | | | | | Older | | | | | |
| Scenario | Easy RT | Hard RT | Easy % error | Hard % error | P. cost | Z cost | Easy RT | Hard RT | Easy % error | Hard % error | P. cost | Z cost |
| A | 641 (75) | 724 (95) | 0 (0) | 0 (1) | 13 (5) | 1 (0.21) | 644 (75) | 752 (103) | 0 (0) | 1 (3) | 17 (6) | 1.13 (0.19) |
| B | 641 (75) | 725 (96) | 0 (0) | 0 (2) | 13 (5) | 1 (0.21) | 662 (79) | 766 (108) | 0 (0) | 1 (5) | 15 (6) | 1.04 (0.21) |
| C | 587 (74) | 648 (92) | 0 (0) | 1 (3) | 10 (4) | 0.82 (0.23) | 623 (75) | 698 (93) | 0 (0) | 0 (2) | 12 (5) | 0.94 (0.21) |
| D | 608 (76) | 690 (98) | 0 (0) | 0 (1) | 14 (5) | 1 (0.21) | 650 (75) | 733 (96) | 0 (0) | 0 (2) | 13 (5) | 1 (0.21) |
|  | d=1.1 | | | | | | | | | | | |
|  | Younger | | | | | | Older | | | | | |
| Scenario | Easy RT | Hard RT | Easy % error | Hard % error | P. cost | Z cost | Easy RT | Hard RT | Easy % error | Hard % error | P. cost | Z cost |
| A | 642 (74) | 725 (95) | 0 (0) | 0 (2) | 13 (5) | 1 (0.22) | 643 (77) | 758 (107) | 0 (0) | 1 (4) | 18 (6) | 1.16 (0.19) |
| B | 644 (74) | 728 (95) | 0 (0) | 0 (1) | 13 (5) | 1 (0.21) | 667 (80) | 778 (110) | 0 (0) | 3 (7) | 17 (6) | 1.05 (0.21) |
| C | 589 (71) | 649 (87) | 0 (0) | 1 (3) | 10 (4) | 0.81 (0.22) | 638 (72) | 719 (92) | 0 (0) | 0 (1) | 13 (5) | 0.98 (0.21) |
| D | 610 (72) | 692 (91) | 0 (0) | 0 (2) | 14 (5) | 1 (0.21) | 666 (76) | 749 (96) | 0 (0) | 0 (2) | 12 (5) | 1 (0.21) |
|  | d=1.4 | | | | | | | | | | | |
|  | Younger | | | | | | Older | | | | | |
| Scenario | Easy RT | Hard RT | Easy % error | Hard % error | P. cost | Z cost | Easy RT | Hard RT | Easy % error | Hard % error | P. cost | Z cost |
| A | 642 (75) | 726 (96) | 0 (0) | 0 (2) | 13 (5) | 0.99 (0.21) | 641 (76) | 767 (109) | 0 (0) | 2 (5) | 19 (7) | 1.21 (0.18) |
| B | 643 (73) | 726 (94) | 0 (0) | 0 (2) | 13 (5) | 1 (0.21) | 679 (81) | 799 (114) | 0 (0) | 4 (9) | 17 (6) | 1.07 (0.2) |
| C | 588 (71) | 649 (88) | 0 (0) | 1 (2) | 10 (4) | 0.82 (0.23) | 652 (76) | 737 (97) | 0 (0) | 0 (2) | 13 (5) | 1.01 (0.21) |
| D | 607 (73) | 690 (93) | 0 (0) | 0 (2) | 14 (5) | 0.99 (0.21) | 686 (73) | 769 (94) | 0 (0) | 0 (2) | 12 (5) | 0.99 (0.21) |

**Supplementary Material C: Combining scenarios**

In the main text, we dissociate four different possible underlying sources of slower reaction times in older adults: A) domain specific deficit, B) general slowing, C) changes in response caution, and D) changes in non-decision time. Though there is evidence that these parameters are generally independent, it is possible aging might affect multiple aspects of cognition. Indeed, in the main text we cite evidence that changes in both response caution and non-decision time are commonly observed in aging. This complicates the ability to use heuristics to identify sources of slowing, as certain combinations may have unpredictable effects on the behavioural costs (depending on the relative size of each effect).

To illustrate this, we ran four additional simulations in which we imposed a large effect (d=0.8) in two of the scenarios identified in the main text. These combinations were:

- A & C: Older adults have both a domain specific deficit and strategic slowing (a change in response caution)
- B & C: Older adults have both general slowing and strategic slowing
- C & D: Older adults have both strategic slowing and increased non-decision time
- B & C: Older adults have both general slowing and increased non-decision time.

The first two combinations were chosen for illustration because the two scenarios have different effects on the behavioural costs. The result of a domain specific deficit and general slowing is to increase RT costs and error costs, whereas a change in response caution increases RT costs and *decreases* error costs. The third combination was chosen because it has been observed in the literature. The fourth was chosen as it has some intuitive appeal – if aging results in some general slowing of brain operations, one might reason that it should affect both decisional and ‘non-decisional’ processes equally.

With the exception that we only simulated a single effect size, the same parameters and procedures were used as in the main text. We omit the proportional cost for legibility. For aid interpretation, we annotate one of the plots (Figure C1) before presenting all four (Figure C2). The solid lines in each plot are the same data as plotted in the main text. We replot these as a reference, so that we can see whether there is a difference in the costs when two scenarios are combined relative to one scenario in isolation. The reference scenario is indicated by the first letter in the subplot title. In the example, (A & C) we plot as reference the data from scenario A (a domain specific deficit). The crosses show the costs from the combined simulations. In the case of our example, combining a large effect in response caution with a large domain specific effect results in a lower error cost (the red cross is below the red line). In contrast, the combination results in higher raw and z-transformed RT costs (the blue and black crosses are above the blue and black lines).


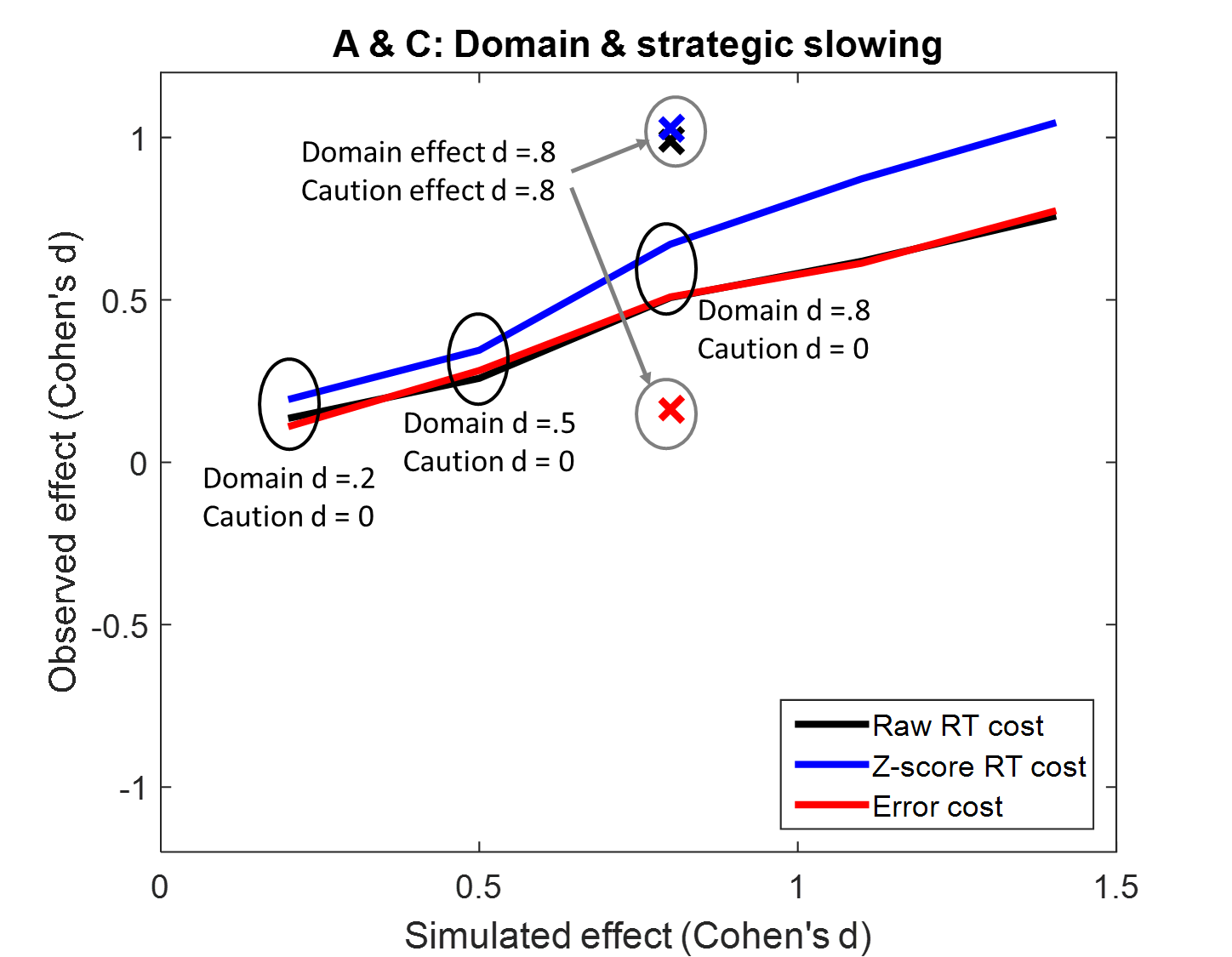


Figure C1. Relationship between the effect size in drift-diffusion model parameters (x-axis) and effect size observed in the behavioural measures derived from the simulated data (y-axis). The lines represent the costs observed in the case of a domain specific deficit only, replotted from Figure 2A in the main text. The crosses represent costs observed where we simulated a large (d=0.8) in both a domain specific deficit and response caution. The red cross is below the red line at the corresponding point on the x-axis, indicating that smaller group differences in error costs are observed when combining a domain specific deficit with response caution compared to a domain specific deficit alone. In contrast, the blue and black crosses are above the respective lines, indicate that RT costs are increased when strategic slowing is combined with a domain specific deficit.

Turning now to Figure C2, where we present the results for all the combined scenarios. The combination of response caution with general slowing produces similar effects to that of a domain specific deficit and response caution (decreased error costs, increased RT costs). In contrast, the lower two panels of Figure C1 show combined changes in caution and general slowing respectively with increased non-decision time in older adults. The addition of non-decision time to these two factors does not alter the data patterns, as RT costs, z-scores and error costs are insensitive to changes in non-decision time. The proportional RT cost, (not shown in the plot) which is sensitive to the mean RT, is slightly reduced in these scenarios. Descriptive statistics are shown in Table C1.

Figure C2. Relationship between the effect size in drift-diffusion model parameters (x-axis) and effect size observed in the behavioural measures derived from the simulated data (y-axis). Crosses indicate results from a single effect size in two parameters in combination. Lines are the results from the reference scenario (the first letter in each combination) replotted from Figure 2 in the main text. If the cross lies on or close to the line, this indicates that the combining the scenarios does not affect the data patterns relative to the scenario in isolation. Positive effect sizes on the y-axis indicate larger costs in the older adult group.

Table C1. Descriptive statistics for simulation of combined scenarios.

|  | Young adults | | Older adults | |
| --- | --- | --- | --- | --- |
|  | Mean RTs (ms) | | | |
| Scenario | Easy | Hard | Easy | Hard |
| A & C: Domain & strategic | 638 (70) | 706 (100) | 672 (73) | 793 (120) |
| B & C: Gen. Slow & strategic | 639 (68) | 706 (97) | 695 (80) | 813 (128) |
| C & D: Strategic & Non-decision | 586 (70) | 653 (101) | 663 (73) | 758 (113) |
| B & D: Gen. slow & Non-decision | 619 (70) | 708 (106) | 678 (80) | 791 (125) |
|  | Mean errors (%) | | | |
|  | Easy | Hard | Easy | Hard |
| A & C: Domain & strategic | 1 (2) | 6 (5) | 1 (2) | 5 (5) |
| B & C: Gen. Slow & strategic | 1 (3) | 6 (6) | 1 (2) | 7 (6) |
| C & D: Strategic & Non-decision | 1 (2) | 6 (5) | 1 (1) | 4 (4) |
| B & D: Gen. slow & Non-decision | 1 (1) | 4 (4) | 1 (2) | 7 (6) |
|  | Raw costs | | | |
|  | RT costs | Error costs | RT costs | Error costs |
| A & C: Domain & strategic | 68 (43) | 4 (4) | 121 (65) | 5 (4) |
| B & C: Gen. Slow & strategic | 67 (43) | 4 (4) | 119 (64) | 6 (5) |
| C & D: Strategic & Non-decision | 68 (44) | 4 (4) | 95 (57) | 3 (3) |
| B & D: Gen. slow & Non-decision | 89 (52) | 3 (3) | 113 (61) | 6 (5) |
|  | Transformed costs | | | |
|  | P. cost | Z. cost | P. cost | Z. cost |
| A & C: Domain & strategic | 10 (6) | 0.45 (0.15) | 18 (9) | 0.59 (0.14) |
| B & C: Gen. Slow & strategic | 10 (6) | 0.45 (0.15) | 17 (8) | 0.54 (0.14) |
| C & D: Strategic & Non-decision | 11 (7) | 0.44 (0.15) | 14 (8) | 0.5 (0.15) |
| B & D: Gen. slow & Non-decision | 14 (8) | 0.49 (0.15) | 16 (8) | 0.53 (0.14) |

**Supplementary material D**

In this section, we describe the data generation for Figure 3 in the main text, which plots the relationship between boundary separation (different coloured lines), drift rates (x-axis), mean RT (solid lines, left axis) and the standard deviation of RTs (dashed lines, right axis) in panel A. Similar plots 3A with a greater range of values can be seen in Ratcliff et al. (2000), though plotted separately for the means (their Figure 4) and standard deviations (their Figure 13). In Figure 3B, we plot the relationship between average drift rates and both RT costs (solid lines, left axis) and z-score RT costs (dashed lines, right axis). In Figure 3B, the average drift rate on the x-axis reflects the average of the drift rates for easy and hard conditions, which always differed by .17.

To construct Figure 3, we simulated data from the drift diffusion model for participants performing a choice RT task with an easy and hard condition (500,000 trials each). We simulated eight individuals at each level of boundary separation, using the same values used for young adults and the five older adult effect size scenarios in our previous simulations (see the second row of Table 1), as well as one standard deviation above and below the maximum and minimum values. For the easy condition, the drift rate values used were (.55, .48, .466, .445, .424, .403, .382, .312). For the hard condition, we subtracted .17 from drift rates in the easy condition. The range of boundary separation values corresponds to one standard deviation above and below the mean value (.155). Non-decision time was fixed at 490ms, between-trial variability in drift rates (η) was fixed to .1. Note that while group differences in non-decision time would also affect the relationship between the mean and standard deviation (c.f. Schmiedek, Lövdén & Lindenberger, 2009), it affects both conditions equally, and is subtracted out when calculating the z-cost.

We also plot the relationship between the mean RTs and standard deviations in Figure D1, to illustrate that the relationship is approximately linear (see also Wagenmakers, Grasman, & Molenaar, 2005).

**
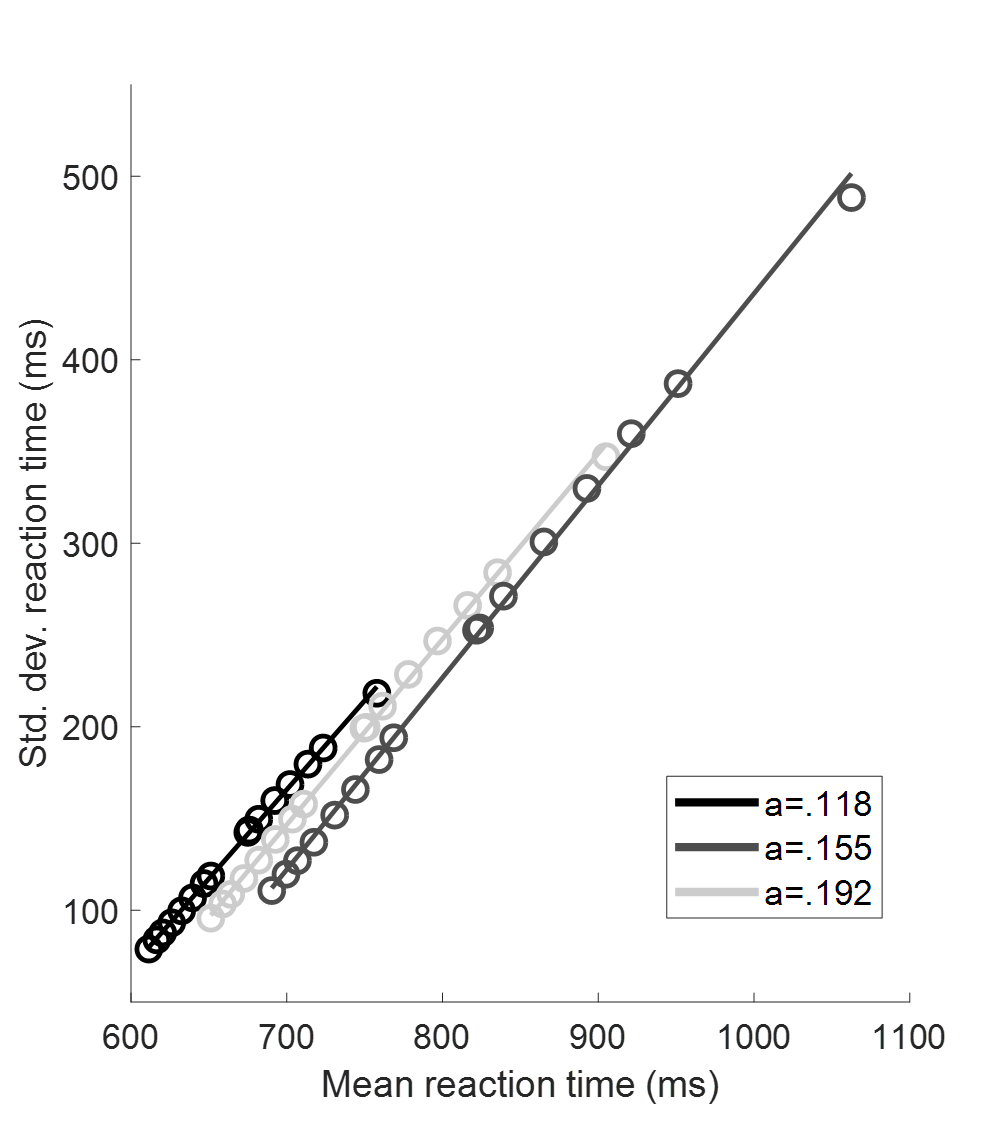
**

Figure D1. Approximately linear relationship between the mean and standard deviation of RTs produced by the DDM using the simulated parameter values. Data points correspond to those used in Figure 3A in main text (see also Wagenmakers, Grasman, & Molenaar, 2005).

**References**

Brown, S. D., & Heathcote, A. (2008). The simplest complete model of choice response time: linear ballistic accumulation. *Cognitive Psychology, 57(3),* 153-178. doi: 10.1016/j.cogpsych.2007.12.002

Donkin, C., Averell, L., Brown, S., & Heathcote, A. (2009). Getting more from accuracy and response time data: methods for fitting the linear ballistic accumulator. *Behavior Research Methods, 41(4),* 1095-1110. doi: 10.3758/BRM.41.4.1095

Donkin, C., Brown, S. D., & Heathcote, A. (2009). The overconstraint of response time models: Rethinking the scaling problem*. Psychonomic Bulletin & Review, 16(6)*, 1129-1135. doi: 10.3758/Pbr.16.6.1129

Ratcliff, R., Spieler, D., & McKoon, G. (2000). Explicitly modeling the effects of aging on response time. *Psychonomic Bulletin & Review, 7(1)*, 1-25. doi: 10.3758/Bf03210723

Schmiedek, F., Lövdén, M., & Lindenberger, U. (2009). On the relation of mean reaction time and intraindividual reaction time variability. *Psychology and Aging*, 24, 4, 841-857.

van Ravenzwaaij, D., & Oberauer, K. (2009). How to use the diffusion model: Parameter recovery of three methods: EZ, fast-dm, and DMAT. *Journal of Mathematical Psychology, 53(6),* 463-473. doi: 10.1016/j.jmp.2009.09.004
